# Removal of a Foreign Body (Pin) from the Throat of a Child

**Published:** 1893-05

**Authors:** A. B. Thrasher

**Affiliations:** Cincinnati


### Removal of a Foreign Body (Pin) from the Throat of a Child.

A case reported to the Academy of Medicine, April 3, 1893,

BY A. B. THRASHER, M.D., CINCINNATI.

About two years ago a little girl six years of age was presented to me for treatment. Her voice was very hoarse, and there was a marked projection over the region of the larynx in front similar to that of a man; this was tender to the touch.

*History:* About two years before I saw her, she was playing, and suddenly began to cry and said she had swallowed a pin. A physician was called, but nothing was discovered. Later she began coughing severely, and was examined by a laryngologist. The physician said he saw very distinctly a pin in the right bronchial tube (?). I suppose he had a very excellent laryngoscope, or his vision was at fault. In the course of four or five months the child grew some better, even after this diagnosis. Within the next four or five months she grew worse, and had some difficulty in swallowing. At this time she had an attack of follicular tonsilitis, after which she grew rather worse, until she was presented to me.

Her phonation was at times greatly impaired, so that it was with great effort she could speak. This was variable, but her general condition grew steadily worse; a dry cough had existed for some time, and it was so irritating that sleep was interfered with. I examined her with a laryngoscope, and found a pin in the larynx. The pin lay across the antero-posterior diameter of the larynx, just above the right ventricular band. Of course, I could not say in which direction the point or head of the pin lay. From nervousness and fright she would not again open her mouth. She was then placed on a lounge and some chloroform given. I introduced my finger as a guide, and with the lateral Fauvel forceps easily removed the pin. The head was sunken under the base of the epiglottis, and had crossed over to the left ventricular band, the point penetrating the right ary-epiglottic fold.

A case quite similar to the above was reported in 1887, by Dr. Langmaid, of Boston, who states that a lady came to him with a history of having swallowed a pin some three months before. He, with another expert laryngologist, examined the patient, and he treated her for some time. Afterward he pulled a pin out from the ventricular band. His opinion was that, in the first place it was not there, but had worked its way there from some other point.

One practical point in the case I have reported this evening is that the physician first called could have easily detected the pin with the finger without any laryngoscope, although the finger should be used with care, from the fact that the foreign body might be pushed further in. From the position of the pin it would have been very difficult to have dislodged it. Being rather a long pin the point probably projected through the ary-epiglottic fold. I heard from the father some three months after the operation, saying that the throat was better and the swelling had mostly disappeared from the larynx.

